# How public confidence was established during the COVID-19 pandemic by Chinese media: A corpus-based discursive news value analysis

**DOI:** 10.3389/fpubh.2022.1012374

**Published:** 2022-10-25

**Authors:** Cheng Chen, Renping Liu

**Affiliations:** ^1^School of Foreign Languages, Zhejiang Gongshang University, Hangzhou, China; ^2^School of Statistics and Mathematics, Zhejiang Gongshang University, Hangzhou, China

**Keywords:** COVID-19 pandemic, Chinese media, news values, discourse analysis, public confidence

## Abstract

During the COVID-19 pandemic, Chinese media played a significant role in dispelling the public panic, establishing the public confidence and stabilizing the society during the COVID-19 pandemic. This corpus-based discourse study explored the discursive construction of news values by Chinese media to reveal how the COVID-19 pandemic was packaged and sold to the public to establish confidence in the news reporting. Adopting corpus linguistic method and the Discursive news values analysis (DNVA) framework, this study examines news values through key words, news quotations, and images in the Chinese domestic mainstream media (http://www.people.com.cn/) during two different phases of the pandemic. The results show that during the first pandemic phase (2019.12.27–2020.4.28) when there had been no treatment protocol or understanding of the medical ramifications, Chinese media dominantly constructed political Eliteness through multimodal resources to portray a people-oriented government, a transparent notification mechanism and an immediate response capability to crises, and to give the public psychological support and to cultivate positive attitudes toward the government's policy. This news reporting way exposes the universal trust of Chinese society in the political authorities. During the second phase (2020.4.29–2020.8.31) when the cognition about the COVID-19 virus had been greatly improved and more medical treatment and prevention methods had been developed, the political Eliteness was replaced by medical Eliteness which was more vital to people's safety during the health crisis. We propose actionable recommendations for scholars to use this in-depth DNVA framework to examine the social trend of thoughts during major public health crisis.

## Graphical Abstract

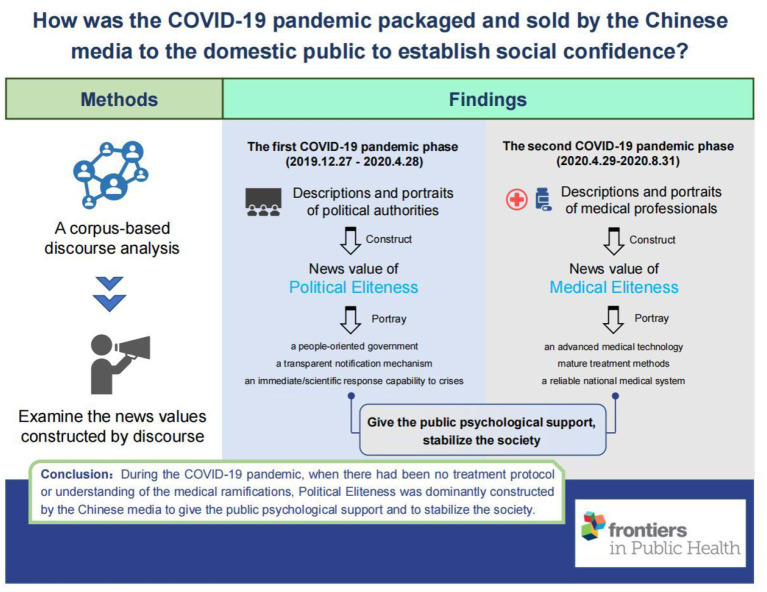

## Introduction

When the COVID-19 pandemic first broke out in the city of Wuhan in China, there was no understanding of the new virus and no treatment protocol. The exponentially increasing patients, the intensive exposure to death, and the breakdown of the medical system caused a great panic among the Chinese public. With the frequent home quarantine and social isolation, the information flow had been cut down and the Chinese-government-dominated mainstream media became the primary channel for the public to learn about the epidemic situation and understand the COVID-19 prevention and treatment methods. The previous studies demonstrated the way in which the health belief education on COVID-19 has directly resulted in the public's mental health concerns [see (1–3)]. The social effect exerted by health belief education is especially strong when it is done through media's reporting and guidance. Moreover, Su et al. (4) examined ways through which legacy media reports on COVID-19 and how social media-based infodemics can result in mental health concerns, and found that media resources should focus on the core issue of how to slow or stop COVID-19 transmission effectively.

During the COVID-19 pandemics in China, as Chinese president Xi Jinping (5) claimed, with strategic ways of information dissemination, Chinese media played a significant role in dispelling the public panic, establishing the public confidence and stabilizing the society. The current study aims to examine how the Chinese media established public confidence by news reporting during different phases of the COVID-19 pandemic.

News values are a set of criteria that determine the selection of what and how is being reported as news (6–8). The examination of news values helps reveal how the media packaged the social event and sold its intended values to the audiences (5). Exposure of the hidden news values within news reports is the very concern of Discursive news value analysis (DNVA), which focuses on how newsworthiness is constructed through multimodal news resources. In this article, we adopt the DNVA method to explore the different news values constructed by Chinese mainstream outlets around the COVID-19 epidemic during different phases (based on the timeline publicized by the Chinese government), as well as the discursive way that the news values are established and sold to the audiences by the Chinese media.

Based on the previous studies (7–9), Eliteness comprises the prominent news values frequently constructed by media. It has been defined as high status or fame (9), which is usually constructed by various status markers such as professional figures and terminology, high-status identities, and their accent/sociolect, recognized names, descriptions of achievement/fame, recognizable key figures, the specialist equipment associated with elite professions, etc. (9). This study focuses on analyzing the construction ways of Eliteness in the Chinese media reporting on COVID-19 pandemic, trying to explore the special presentations of Eliteness and improving the definitions of Eliteness. We hope this study will offer a new investigation of DNVA in COVID-19 news discourse and prove the application potentials of DNVA framework in public health crisis.

## News values and a discursive approach to news values

Emanating from journalism and communications research, the concept of “news values” has been predominantly identified as the impetus for the selection of news content (story/event) (7, 10). For example, Palmer (7) defined news values as aspects of an event which is in accordance with the timeliness, interest, importance, etc. Harrison (11) specified that news events intrinsically carrying news value as “events are deemed to be self evidently newsworthy”. By contrast, linguists such as Bell (8), Richardson (12), Baker et al. (13) expanded the scope of news values beyond the content-based perspective, and interpreted it as a discourse construction process. For example, Bell (8, 14) specified the controlling roles of the news values in constructing news discourses, and also indicated that the language is frequently used to enhance and maximize news values.

The aforementioned journalistic and linguistic perspectives were amalgamated by Bednarek and Caple (15–17), who advanced a discursive approach that portrays news values in two interrelated dimensions. The first one holds that news values are constructed through discourse, which encompasses any discourse that plays a part in the news process, for example, verbal and visual input material, interviews, press releases, etc. (17, 18). Second, news values are defined as a quality of texts which self-expresses through a wide range of semiotic devices (language, image, typography, layout, sound, etc.) (17). Hence, the discursive approach to news values [commonly known as “Discursive news values analysis (DNVA)”] entails the investigation of the multiple semiotic choices utilized in the construction of news discourse in the context of their roles in establishing news values (18). With its capability for systematic analysis of news value construction through a series of multimodal resources, the DNVA framework helps researchers gain a fuller understanding of how the news is forged (19). Since its introduction a decade ago, DNVA has been progressively developed by expanding the scope of resource types that construct news values, from written language (19) to multimodal devices (15, 20, 21). Accordingly, the categories and definitions of news values have also been constantly updated and improved. Whilst they had formerly been limited to 7 categories that solely centered on linguistic devices (15), now they have increased to 11 categories with a broader purview, including not only linguistic but also visual signs. Recently a cross-cultural perspective is adopted and several news values have been redefined and linked with publication time and publication's target audience (9, 22). For example, Proximity, originally defined as “the geographical or cultural nearness of an event or issue” (18), has been redefined as “geographically or culturally near the target audience” (22).

With its essential focus on newsworthiness analysis, the DNVA framework also covers the examination of the whole news communication process, including the motivations behind maximizing news value (e.g., social powers, potential value of events, journalistic value), the construction of news reporting, and the social effect of the news reporting, which is in turn determined by the newsworthiness. For example, Caple et al. (9) interpreted national days in news reporting as closely tied to the history of a nation and the discursive construction of national identity, and estimated the social reaction of the news reporting.

Taking a further step from the “manually-conducted” DNVA (through close reading of relevant texts), researchers [e.g., (22, 23)] have successfully paved the way for the application of corpus-linguistic methodology in DNVA through several case studies. Corpus techniques have been proved to allow researchers to examine the most salient linguistic devices in each corpus which have the potential to construct marked news values, and to provide insights into “how happenings are sold to audiences as newsworthy” (17).

## Adapting discursive news values analysis to Chinese health crisis

As the previous studies have demonstrated, the broadcast of the exact information during the COVID-19 pandemic greatly helps decrease the public fear, stabilize society and improve the medical prevention and treatment [see (24, 25)]. For example, Rahmat et al. (26) proceeded a questionnaire survey to medical students and found specific strategies from the government officials in information broadcast to address medical students' uncertainties and increase the adoption of technology amid the COVID-19 pandemic. Among the existing related studies, media discourse has been proved as the most influential tool to dominate various social factors. For example, Yoosefi Lebni et al. (27) examined how the COVID-19 pandemic affected economic, social, political, and cultural factors, and found that the media supported by the government greatly helped to build confidence among people, overcome their fear, anxiety, stress, and mental health problems. The function of the media was proved as prominently effective in the COVID-19 pandemic in China [e.g., (25, 28, 29)].

As DNVA has a powerful framework for systematic analysis of news value construction through multimodal resources, it has been frequently adapted in exploring the way by which the media (or journalists) package a health crisis and the social effect of the news reporting, especially the COVID-19 pandemic (15). For example, Langbecker et al. (30) examined the news values construction by comparatively analyzing the journalistic coverage of the National Health System (SUS) by “Folha de São Paulo,” and the National Health System (SNS) by “El Pais.” Building on a discursive view on news values, Andersen et al. (31) outline how news values are discursively constructed through online news headlines on health topics from three Nordic countries, and examine how journalists construct their target audience discursively by imposing problems and projecting desires for action and change onto readers. Colak (32) analyzed the news values of social media regarding the term, COVID-19, with the phenomenological method. Trishchuk et al. (33) analyzes the newsworthiness of online media during the crisis caused by the coronavirus pandemic and highlights changes in the content of media platforms during the spread of the coronavirus pandemic.

Though DNVA has been primarily applied to Western-centric languages and contexts (9), variations in the construction of newsworthiness in the news reporting of Western and Chinese media have been more frequently compared in recent studies. For example, Zhang and Caple (23) examine news values in four news outlets from China, Britain, Australia and the United States in their English-language news reporting about the Chinese tennis player, Li Na. Caple et al. (9) present a cross-linguistic comparison of news values in “national day” reporting from China and Australia. However, these previous China-Western comparative studies were actually still Western-centric, as they focused on Western-interested stories [for example, the individualism of Li Na (23), the democracy of nationalism (9)] and aimed at exploring how Chinese newsworthiness deviates from Western values, instead of analyzing China's media communication in terms of Chinese stories. This study focused on analyzing Chinese news discourse and exploring the distinctive news process and the ideological forces that drive the Chinese media in reporting China's health crises. In this way, the present study makes the first attempt at a China-centric news story analysis.

## Data and methods

### Data

In this study, we adopt the COVID-19 pandemic phases categorized by Chinese national government which specifically describe the pandemic situations in China (We did not adopt the WTO phases as they portray the pandemic situations of the whole world). As reported by the National Health Commission of China, based on the affected area and the prevention and control mechanism, the COVID-19 pandemic in China can be divided into two general phases (34). The first phase is from December 27th, 2019 to April 28th, 2020 when the COVID-19 pandemic first occurred in the city of Wuhan, rapidly spread nationwide, and the emergency response was explored and launched. The second phase is after April 29th, 2020, in which the epidemic has been generally controlled and the regular prevention measures are being implemented. Therefore, this study established two Chinese news corpora to analyze the different news values constructed by Chinese media in different COVID-19 phases.

The world has become a global village and technology use has made it a smaller world through online media (35). This original study chose online news reporting from “*people.cn*” (http://www.people.com.cn/) to establish the corpus, which are the Chinese-government-dominated mainstream online newspapers and enjoy the widest domestic circulation with extensive readership. The Chinese word “新冠肺炎(COVID-19)” was used as the search term to retrieve Chinese news reports around the COVID-19 pandemic during the two periods: from December 27th, 2019 to April 28th, 2020 (the first phase); and from April 29th, 2020 to August 31th, 2020 (the second phase). Both of selected periods are equal in duration of time. For each period, 200 related news reports were collected in random to establish the corpus (the two corpora were marked as Corpus-Phase 1 and Corpus-Phase 2). Meanwhile, for retrieving the keyword lists of Corpus-Phase 1 and Corpus-Phase2, another 200 news reports with no specific theme before December 27th, 2019 (from August 26th, 2019 to December 26th, 2019) were collected as the reference corpus. The Chinese texts of all the three corpora have been annotated and segmented into Chinese words by the word segmentation tool “pkuseg (https://github.com/lancopku/PKUSeg-python).”

Considering that the news reports are usually of different lengths, which renders the two corpora uneven in word counts (Table 1), the analysis of this study is based on percentages instead of the total numbers, as the statistical techniques demonstrated in the previous studies (36, 37). As the present study is concerned with how news values are discursively constructed through both language and images, a total of 54 photographs associated with the verbal stories were collected in Corpus-Phase 1 and 62 photographs were collected in Corpus-Phase 2 (Table 1). As photographs of the two corpora are not equal in number, the analysis of the news values constructed by visual resources is also based on percentages, for ensuring the comparability.

**Table 1:** The two corpora of Chinese media and Indian media.

**Corpus**	**Corpus-phase 1** **(2019.12.27-2020.4.28)**	**Corpus-phase 2** **(2020.4.29-2020.8. 31)**	**Reference corpus** **(2019.8.26-2019.12.26)**
The number of news reports Total character count	200 276,844	200 279,648	200 119,190
The number of photographs	54	62	Not collected

### Methods

This DNVA study combines corpus linguistic techniques to examine the news values construed: ① in keywords and their concordances, revealing the focus of the reporting (38); ② in the identities of the quoted speakers and the frequencies they have been quoted, reflecting the media's selection of the values delivered by these quotations (39); and ③ in the photographs illustrated with the news reports, offering insights into how “COVID-19” is presented to audiences visually (40). These techniques provide statistic support for analyzing the way that news values are constructed (41), including examining the “news actors,” “happenings” and “issues” (15). The procedures of this study are comprised of four steps as follows.

First, the corpus analysis tool AntConc 3.5.8w was used to conduct the keywords analysis, helping ascertain which Chinese words demonstrate saliency in the two news corpora (Corpus-Phase 1 and Corpus-Phase 2), respectively. As keywords works as a “pointer” to the construction of certain news values (17), this focus revealed the news values established by the Chinese media and the discrepancies during different two COVID-19 Phases. The concordance lines of the keywords were further examined to better observe the way that the news values are actually constructed.

Second, we examined the quotations in the news discourse manually, including analyzing the identities of the quoted speakers and counting the frequencies of the quotations, to identify the values that the media intended to sell in selection of social voices (21).

Third, in order to determine the way in which news values are constructed in imagery, we labeled the photographs in terms of their camera techniques and content, which includes the visual participants, their activities, the circumstances where these activities take place, etc. (40). On the other hand, camera technique comprises “shutter speed (how fast), aperture (how much light), focal length (how much in focus), lens (how distorted/natural/condensed the shot), and angle (how high or low the angle)” (15).

Fourth, the features of the multimodal semiotic resources centered on the above three aspects have been further analyzed in the DNVA framework, to examine how they work together to emphasize the newsworthiness of the COVID-19 epidemic events. The 11 news value categories (42) were adopted as the criteria to define and interpret the news values, as these 11 categories define news values both for linguistic and visual resources (43). Moreover, the social effect of the news reporting upon the public around the COVID-19 epidemic has been further discussed based on the newsworthiness constructed by the Chinese media.

## Findings and analysis

### Keywords in reporting and the construction of news values

With AntConc 3.5.8w, these two Chinese corpora were compared reciprocally and two keywords lists were obtained, one with 46 words for Corpus-Phase1 and the other with 31 words for Corpus-Phase1 (see Table 2). These keywords were classified into different groups based on their semantic domain, by which it can be inferred what the pandemic event is mainly constructed as. The semantically categorized Chinese keywords, their English translations, the frequencies and the keyness statistics are listed in Table 2. The differences can be easily found in the two corpora.

**Table 2:** The keywords in reporting of the COVID-19 pandemic during different phases by Chinese mainstream media.

**Semantic domain**	**Corpus-phase 1**	**Corpus-phase 2**	**News value**
Common citizens and their health	*患者* (patient 225/137.57) *病例* (case of illness 74/53.37) *公共卫生* (public health 186/134.19) *生命安全* (safety 119/85.83) *身体健康* (health 115/82.95) *生活必需品* (daily necessities 50/36.06) *群众* (the citizens 351/78.43) *人民* (the people 468/58.41) *收治* (receive and cure 77/55.53) *救治* (treat and cure 293/192.27) *复工复产* (work resumption 201/145.02)	*患者* (patient 232/138.77) *病例* (case of illness 116/81.78) *老年人* (old people 105/74.02) *救治* (treat and cure 126/73.08) *生命安全* (safety 56/39.47)	Personalisation
Political leaders/institutions and their actions	*各级党委* (Party committees at all levels 100/57.21) *习近平* (President Xi Jinping 842/52.72) *李克强* (premier of the State Council 760/48.67) *领导* (leaders 210/40.57) *干部* (officials 168/32.11) *党中央* (the Central Committee of the CPC 269/26.96) *领导小组* (leading group 101/25.02) *加强* (reinforce 758/199.31) *保障* (guarantee 403/110.69) *联防联控* (Joint Prevention and Control Mechanism 148/106.76) *坚决* (resolute 253/87.27) *强调* (emphasize 348/87.04) *确保* (ensure 201/78.04) *统筹* (unified planning 200/74.31) *落实* (implementation 292/54.92) *抓好* (vigorously undertake 134/54.2) *加大* (enhance 75/54.09) *抓实* (accountability for actions 74/53.37) *全力* (spare no effort 114/52.69) *组织* (organize 230/49.73) *控制* (control 68/49.04) *指出* (point out 303/48.55) *指示* (instruct 59/25.69) *集中* (centralize 124/48.19) *部署* (deploy 250/48.15)		Eliteness
War metaphor	*阻击战* (the battle for blocking the enemies 170/122.64) *打赢* (win the battle 196/93.01) *攻关* (storm a strategic pass 117/63.5) *斗争* (fight 115/57.49) *人民战争* (the people's war 66/47.6) *抗击* (beat back 65/46.87) *战胜* (triumph over 107/38.21) *总体战* (the battle by mobilizing all resources 53/38.22) *保卫战* (the battle for defense 38/27.4)	*斗争* (fight 119 /58.33) *人民战争*(the people's war 36/25.37) *战胜* (triumph over 81/22.71) *战役* (the battle 36/25.37)	
Professional medical research and treatment	*科学* (science 185/104.26) *科研* (scientific research 134/61.45)	*病毒* (virus 193/136.1) *冷链* (cold train 183/129.04)	
		*中医药* (traditional Chinese medicine 151/106.47) *核酸检测* (nucleic acid testing 148/104.35) *科学* (science 170/91.49) *常态化* (Normalization 123/77.51) *医疗* (medical treatment 208/62.16) *接种* (vaccinate 87/61.33) *发热* (fever 81/57.1) *隔离* (quarantine 81/57.1) *消毒* (sterilize 80/56.39) *口罩* (medical facemasks 75/52.87) *门诊*(section for outpatients 85/51.45) *中西医* (traditional Chinese medicine and western medicine 64/45.11) *预防* (prophylactic 64/45.11) *流调* (epidemiological investigation 37/26.08) *外防* (prevent the coronavirus from entering 39/27.49) *消杀* (sterilize 36/25.37) *重症* (patients in severe condition 36/25.37) *疫苗研发* (vaccine development 32/22.55) *医学观察* (medical observation 33/23.26) *疾控中心* (Center for Disease Control 33/23.26)	Eliteness

The keywords of Corpus-Phase1 focus on depicting three aspects of semantic domains: ① the political leaders/institutions and their actions against the COVID-19 pandemic, ② the common citizens and their health, and ③ the war metaphors for the combat against COVID-19. The first semantic domain is mainly composed of the names of the political leaders (for example, 习近平 [Chinese President Xi Jinping], 李克强 [Chinese Premier Li Keqiang]), the leadership (for example, 领导小组 [leading group], 党中央 [the Central Committee of the CPC]) and the leaders' political actions (for example, 加强 [reinforce], 控制 [control], 指示 [instruct]), portraying the organizing, planning and supervising actions of the political leaders/institutions against the COVID-19 pandemic, consequently construing the news value of Eliteness (see EXAMPLE 1 and EXAMPLE 2).

#### Example 1

**English translation:** The National Health Commission will further reinforce the links between governmental departments and learn specific measures to jointly enhance epidemic prevention and control.

(*people.cn*, 2020-01-19)

#### Example 2

**English translation:** Chinese President Xi Jinping made important instructions on the COVID-19 pandemic, emphasizing that people's safety and health should be put first and the epidemic would be resolutely controlled. Chinese Premier Li Keqiang made concrete instructions.

(*people.cn*, 2020-01-20)

The keywords of the second semantic domain mainly illustrate the identities of the common individuals (for example, 患者 [patient]) and the benefits that they can enjoy, such as medical care, necessities of life, convenient transportation (as shown in EXAMPLE 3 and EXAMPLE 4), construing the newsworthiness of Personalisation. The concordance lines further demonstrate that the individuals' identities and their benefits are usually illustrated in the political leaders' or political institutions' statements as the government's policies/plans are geared to benefit people. For example, the medical benefits offered to the patients illustrated in Example 3 were issued as a policy by “Li Keqiang (Chinese Premier)” in the leading group conference (see EXAMPLE 5); the daily supply offered for common citizens during the COVID-19 pandemic were promised by “the Ministry of Finance and the National Medical Insurance Administration” as an emergency notice (see EXAMPLE 6). Therefore, it can be argued that the establishment of Personalisation is accompanied by the construction of Eliteness.

#### Example 3

**English translation:** For ensuring that the patients diagnosed as COVID-19 infection can all afford to have the treatment, the Ministry of Finance and the National Medical Insurance Administration jointly issued an emergency notice, proposing that the drugs and medical services for the diagnosis and treatment of COVID-19 infection can be temporarily included in the payment scope of medical insurance fund.

(*people.cn*, 2020-01-23)

#### Example 4

**English translation:** We should ensure the normal supply of daily necessities, coordinate and ensure the supply of vegetables in key epidemic areas, maintain smooth transportation, give priority and free access to vehicles carrying out emergency transportation tasks, ensure the key supply of coal, electricity, oil and gas, and make every effort to prevent and control the epidemic.

(*people.cn*, 2020-01-29)

#### Example 5

**English translation:** Li Keqiang, member of the Standing Committee of the Political Bureau of the CPC Central Committee, Premier of the State Council, and the leader of the central leading group for responding to the COVID-19 infection, presided over the leading group conference and pointed out: For ensuring that the patients diagnosed as COVID-19 infection can all afford to have the treatment, the Ministry of Finance and the National Medical Insurance Administration jointly issued an emergency notice, proposing that the drugs and medical services for the diagnosis and treatment of COVID-19 infection can be temporarily included in the payment scope of medical insurance fund.

(*people.cn*, 2020-01-23)

#### Example 6

**English translation:** The Ministry of Finance and the National Medical Insurance Administration jointly issued an emergency notice: We should ensure the normal supply of daily necessities, coordinate and ensure the supply of vegetables in key epidemic areas, maintain smooth transportation, give priority and free access to vehicles carrying out emergency transportation tasks, ensure the key supply of coal, electricity, oil and gas, and make every effort to prevent and control the epidemic.

(*people.cn*, 2020-01-29)

The keywords as war metaphors compare the COVID-19 pandemic prevention and efforts with a battle, thereby overstating the lethality of the virus, emphasizing the emergency to eliminate the virus, and consequently enhancing the people's will to fight. In this way, the newsworthiness of superlativeness has been constructed. On the other side, the metaphor of war imbues the COVID-19 pandemic prevention and control with political hue, which is in accord with the dominant keywords on “Political leaders/institutions and their actions.”

On top of the three main semantic domains discussed above, there are two keywords (科学[science] and 科研[scientific research]) conveying the abstract concepts for “professional medical research and treatment.”

Comparatively, in Corpus-Phrase2, the keywords around “professional medical research and treatment” take the dominant semantic domain and express much more concrete medical concepts, such as 中医药 (traditional Chinese medicine), 流调 (epidemiological investigation), 疫苗研发 (vaccine development), etc. The concordance lines further show that these medical keywords are closely associated with medical elite professions; for example, the keyword “疫苗研发 (vaccine development)” occurred with an abundance of specialized/technical terminologies and the identities of medical experts, thus construing the news value of Eliteness (see EXAMPLE 7). Besides, Corpus-Phase 2 also contains keywords depicting “common citizens and their health” and “war metaphor,” but is much less deficient in quantity and types than those of Corpus-Phase 1.

#### Example 7

**English translation:** Huang Luqi, an academician at the Chinese Academy of Engineering, specifically introduced that “the scientific research team has always insisted on giving top priority to vaccine research and development, and is committed to developing safe, effective and accessible vaccines”; “We will always adhere to the scientific law and simultaneously promote the five technical routes of inactivated vaccine, recombinant protein vaccine, adenovirus vector vaccine, attenuated influenza virus vector vaccine and nucleic acid vaccine, so as to maximize the success rate of vaccine research and development.

(*people.cn*, 2020-07-31)

Generally speaking, during the two COVID-19 pandemic phases, the Chinese media dominantly constructed the news value of Eliteness, albeit in different ways. During the first phase, the Chinese media mainly establishes Eliteness through the authoritative identities of the political leaders/institutions and their actions (including the war concepts). It can be seen that when the pandemic first occurred and people had no understanding of the medical ramifications, the Chinese media made full use of the political authoritativeness to establish people's confidence and stabilize the society (the Eliteness which is constructed by political authorities is referred as Political Eliteness in the following part). Moreover, the news value of Personalisation has also been highlighted to establish the common people's positive attitude toward the government's policy; Superlativeness has been construed by war metaphor to create an atmosphere of emergency, which further reinforce people's confidence. In contrast, during the second phase when the cognition about COVID-19 virus had been greatly improved and more medical treatment and prevention methods had been developed (the Eliteness which is constructed by medial profession is referred as Medical Eliteness in the following part), the political Eliteness had been entirely replaced by the medical Eliteness with more technical terms and medical authoritative identities. In this way, people's confidence has been constructed by the reporting on the medical development.

### News values constructed by quotations

As news has been defined as “what an authoritative source tells a journalist” (8), quotation is deemed as the most characteristic feature of news language (35, 44). The inclusion of external voices is of crucial interest in news discourse studies (23, 39, 45), and one of the major themes that the scholars are concerned with is the relations between quotation and social power, which were mainly found and interpreted in who gets quoted. For example, van Dijk (39) revealed that in European news discourse, minority groups were quoted much less than the whites, demonstrating the European media showed racial discrimination in reporting. Zhang and Caple (23) inferred the diplomatic relations between China and Japan in the summit meetings by comparing the different frequencies of the quotations from the Chinese and Japanese heads of State in international reporting. This research explores the identities of the quoted speakers and the frequencies they have been quoted, and demonstrates the newsworthiness constructed by the selection of these quotations.

Table 3 shows that the Chinese media reported the COVID-19 pandemic by frequently quoting from the voices made by two groups of people, including “Political elites” and “Professional medical elites” (only 5 instances at most for each identity are listed, due to space limit). The two groups of the speakers both indicate high social, professional or political identities, and so establish the news value of Eliteness. Corpus-Phase 1 dominantly quoted the words of “Political elites,” expressing the governmental policies and plans for preventing and controlling the COVID-19 pandemic (see EXAMPLE 8 and EXAMPLE 9); Corpus-Phase 2 mainly cited the sayings of “Professional medical elites,” specifying the medical prevention and professional treatment methods (see EXAMPLE 10 and EXAMPLE 11). The statistics of this part further verifies the results of the “Keyword part” that political Eliteness dominates the news reporting in the first phase of COVID-19, and medical Eliteness dominates the news reporting in the second phase of COVID-19.

**Table 3:** The identities of the speakers and their concurrences in news quotations.

**Media**	**Corpus-phase 1**	**Corpus-phase 2**	**News value**
**Identity** **of the speaker**			
Political elites	**383 (99%)** Example: *习近平* (Xi Jinping, President of China) *孙春兰* (Sun Chunlan, Vice Premier of the State Council of China) *黄坤明*(Huang Kunming, Deputy Head of Publicity Department of China) *李斌* (Li Bing, Deputy Director of the National Health Commission of China) *贺青华* (He Qinghua, the first-level inspector of the National Health Commission of China)	**8 (2%)** Example: *陈竺* (Chen Zhu, Vice Chairman of the Standing Committee of the National People's Congress) *何维* (He Wei, Vice President of Chinese People's Political Consultative Conference) *徐南平* (Xu Nanping, Vice Minister of Science and Technology Department)	
Professional medical elites	**3 (1%)** Example: *钟南山*(Zhong Nanshan, a prominent Chinese expert in respiratory diseases)	**319 (98%)** Example: *蒋荣猛*(Jiang Rongmeng, member of the NHC expert team) *张伯礼*(Zhang Boli, academician of the Chinese Academy of Engineering) *黄璐琦*(Huang Luqi, an academician of the Chinese Academy of Engineering) *曾益新*(Zeng Yixin, deputy head of the National Health Commission of China) *赵丽君*(Zhao Lijun, Vice Dean of Sichuan Friendship Hospital)	

Bold values indicate occurrences of the identity (proportion of the quotations).

#### Example 8

**English translation:** Sun Chunlan (Vice Premier of the State Council of China) stressed that it is necessary to strictly carry out the territorial responsibility system and the first diagnosis responsibility system, to search for the source of the disease, to block the source infection, to isolate and prevent the proliferation of the COVID-19 virus, to control the source of infection, to block the route of transmission, and to prevent internal proliferation and external export.

(*people.cn*, 2020-01-22)

#### Example 9

**English translation:** President Xi Jinping has made instructions on this regard, stressing the need to put people's lives and health on the first place, and to resolutely contain the spread of the epidemic.

**(***people.cn*, 2020-01-21)

#### Example 10

**English translation:** “In the process of vaccination, the cases of mild fever are < 0.1%, and the incidence of severe adverse reactions, such as anaphylaxis, is about 2 cases per million. These conditions have been treated in a timely manner.” Zeng Yixin (deputy head of the National Health Commission of China) said.

(*people.cn*, 2020-07-31)

#### Example 11

**English translation:** Jiang Rongmeng, member of the NHC expert team, said that recently, sporadic COVID19 cases occurred in many places of China, and the source of infection has not been thoroughly traced back, so there is still a risk of “transmission from objects to human beings.”

(*people.cn*, 2020-07-29)

### News values constructed by photographs

**Figure 1:**
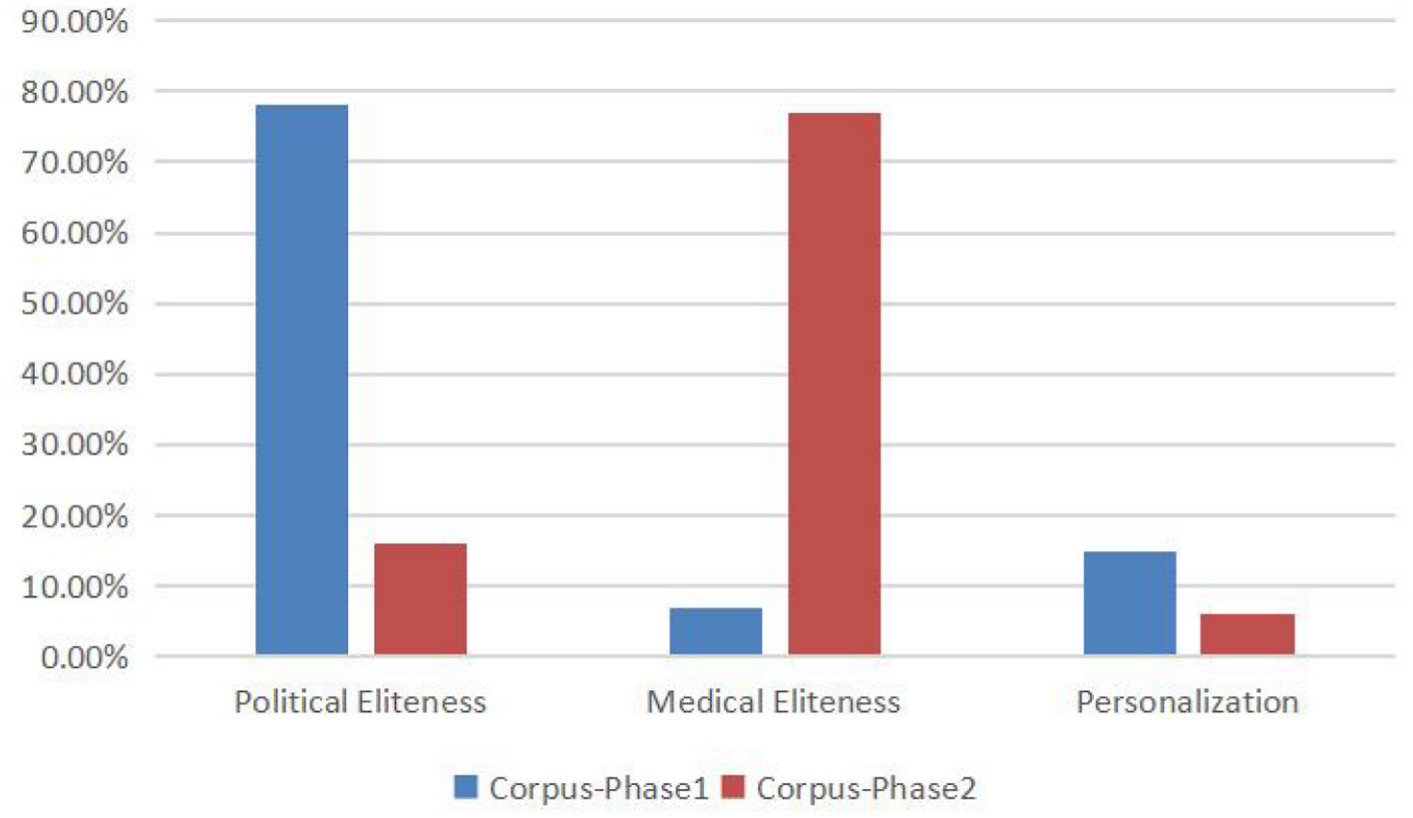
The construal of news values in the photographs in the Chinese media during two different COVID-19 phases (as percentages).

Each photograph from the two Chinese corpora and the news values it constructs have been analyzed from the perspectives of “content” and “camera technique” (25). In order to determine the news values that underpin each photograph, the results of the analysis were collated in an MS Excel spreadsheet as can be seen in Figure 1. It can be seen that the news photographs during two different phases construct utterly different news values.

Figure 1 shows that Eliteness is the commonly dominant news value constructed by Chinese media during the two phases. During the first phase, the portraits of Chinese political top leaders dominate the content of photographs, which are frequently taken as close-ups, with their faces and expressions clearly recognizable and with microphones and platforms indicating powerful rights of speech (for example, the photograph portrays that President Xi Jinping delivered a speech at the conference on organizing COVID-19 prevention work, people.cn, 2020-02-24). Besides, a small part of Chinese political leaders are portrayed in the center of a group of people, in long shot photographs, flanked by subordinates and bodyguards (e.g., the photographs that portray President Xi Jinping visiting Beijing Ditan Hospital, with a group of officials standing behind him with blurred faces, *people.cn*, 2020-02-07).

Comparatively, during the second phase, Eliteness is mainly constructed through visual content by portraits of medical experts. Different from the portraits of political leaders during the first phase, the Chinese media did not depict the medical experts with individual close-ups, but with mid-shots of professional groups (e.g., the photograph captures two doctors in Wuhan provided remote diagnosis and treatment services for aged patients, *people.cn*, 2020-07-08; the group photograph depicts Huami Corporation cooperated with Zhong Nanshan and his medical research team to conduct post-hospital management of COVID-19 patients, *people.cn*, 2020-07-16). Besides, the medical Eliteness are mainly reinforced by professional equipment and academic background (for example, the remote diagnosis and treatment service facilities, *people.cn*, 2020-07-08; the prominent display of medical institutional logo and the academic seminar room, *people.cn*, 2020-07-16).

The news value of Personalisation has been constructed in a comparatively low frequency, through the portrait of the citizens in daily life or doctors and nurses in working (for example, the photograph portrays a cleaner carrying the disinfection work in the subway station, *people.cn*, 2020-01-23). Generally speaking, the news values constructed by news photographs are in accordance with those construed by keywords and quotations, which are dominated by Eliteness of political top leaders during the first phase and by Eliteness of medical experts during the second phase.

## Discussion

This discursive analysis of news values shows how a variety of words and images in Chinese media work together to construct a combination of news values. During the first phase when there was insufficient medical cognition on the virus, the Chinese media employed multimodal resources (both textual and visual) to forge a “political authoritative context model (46)”, which portrayed a people-oriented government, a transparent notification mechanism and an immediate/scientific response capability to crises, and gave the public psychological support and cultivating positive attitude toward the government's policy. During the second phase when the cognition of COVID-19 virus had been greatly improved and more medical treatment and prevention methods had been developed, the “political authoritative context model” was replaced by the “medical specialized context model (46)” which was more vital to people's safety during the COVID-19 pandemic and could more directly give people a sense of security.

It is worth noting that the reason why the Political Eliteness can give the public psychological support and stabilize the society is because the Chinese government did respond efficiently and made effective policies to keep the novel coronavirus largely at bay and save lives. In its fight against the pandemic, China puts people first and follows a people-centered development philosophy to minimize the impact of the pandemic on economic and social development. In the more than 2 years since the COVID-19 pandemic erupted, the Chinese government has developed and implemented a dynamic clearing policy for the whole country, including regular testing and contact tracing, centralized quarantine and the use of big data to prevent the spread of the virus between cities. This strategy suggests a strong policy mix of NPIs and immunization, and an emphasis on avoiding lockdowns. Importantly, it requires local governments to rely on an epidemiology-backed system to respond early to cluster outbreaks and stop the spread of the virus (47). Moreover, the Chinese government has exerted appropriate and competent efforts in order to share information with the public during the pandemic (48). Based on the survey of Hu et al. (49), information disclosure was a top priority for official responses to the COVID-19 pandemic. The effective policing response made people feel they have received timely disclosure and gave them sufficient incentive to implement community prevention and control measures (50–52). Therefore, China's effective governance is the reason for Chinese media to construct Political Eliteness in its news reporting as the dominant news value.

Scholars (53, 54) have demonstrated that public opinion support moderates various negative social factors including job stress, family crisis and people's job turnover intention. Media's reporting, as the most effective guide of the public opinion, exerted the greatest influence on the social stability. The strategic information broadcast of the media is indispensable in dispelling uncertainty, fear, and mental stress to unify global communities in collective combat against COVID-19 disease (55). It can be seen that during the two different pandemic phases, the Chinese media exercises overall control in the production of news discourse, making sure it is coherent in the narratives both textually and visually, and appropriate for constructing public confidence (56).

At the same time, the role of journalists in interpreting news events should be duly noted (39). Indeed, the values underpinning news are not intrinsic to any event, but assigned extrinsically by journalists (19). As Shirk (57), Sun (58) and Chan (59) claimed, China's news institutions are geared toward public service-oriented functions in modern times. In more pointed terms, the journalists working under the auspices of the government have the responsibility to select news stories that meet public expectation, for example, reporting the contents that the people are concerned about, using language with high public acceptance, guiding the public opinion positively, etc. (59). This DNVA study reveals the Chinese media's intentions and ways to establish a positive social context by constructing political and medical Eliteness in presenting a public health story to audiences (19).

## Conclusion

This study is the first attempt to analyze news values in the COVID-19 news discourse, which has brought together corpus linguistic techniques, multimodal discourse analysis and DNVA to examine the ways in which new values are constructed through complex news resources. As noted above, this DNVA study has demonstrated that in facing an unprecedented health crisis such as the COVID-19 pandemic, with the absence of any treatment protocol or understanding of the medical ramifications, political authoritativeness is the first choice by Chinese media to construct public confidence. As Guan (60) pointed out, China is a society advocating the faith in politics, in which people have been educated to believe in the political philosophy (e.g., Marxism) and the political governance. Thus, it can easily be understood that when the health crisis is beyond people's capacity, politics features predominantly in the reporting/media as the spiritual or even supernatural backup.

This study has updated and improved the specifications of news values illustrated by previous DNVA studies. We have found that the same news value can be constructed in different presentations. The dominant news value of Eliteness has been established as Political Eliteness by the media during the first pandemic phase, and established as Medical Eliteness during the second pandemic phase. While most of the previous DNVA studies interpreted the news values in one-fold presentation [e.g., (9, 15–18)].

This study has demonstrated the advantages of DNVA in analyzing media's mental model and behavioral model in the reporting of major health crisis crises. With the capabilities for multimodal discourse analysis, corpus-assisted discourse analysis, and ideological and power relational analysis, this DNVA framework has revealed the systematic process of newsworthiness construction, much more elaborately and clearly than many of the previous analyses of news discourse. Furthermore, news discourse analysis should continue to tell us truths about the values of a society. The news values and their construction ways presented in this essay provide a more in-depth framework to examine the social trend of thoughts during the COVID-19 pandemic. We hope this article has proved the application potentials of DNVA framework in public health crisis and introduced a new approach to social ideological analysis.

This study offers a new investigation of DNVA in COVID-19 news discourse and provides momentum to scholars worldwide who are interested in adopting DNVA to the topic of public health. With its focus on news values in Chinese stories, the paper also contributes to research on Asian context-centered analysis.

## Data availability statement

The original contributions presented in the study are included in the article/supplementary material, further inquiries can be directed to the corresponding author.

## Author contributions

CC took charge of the data analysis and paper writing. RL took charge of the data collection and paper revision. All authors contributed to the article and approved the submitted version.

## Conflict of interest

The authors declare that the research was conducted in the absence of any commercial or financial relationships that could be construed as a potential conflict of interest.

## Publisher's note

All claims expressed in this article are solely those of the authors and do not necessarily represent those of their affiliated organizations, or those of the publisher, the editors and the reviewers. Any product that may be evaluated in this article, or claim that may be made by its manufacturer, is not guaranteed or endorsed by the publisher.
